# The Effect of Dexmedetomidine as a Sedative Agent for Mechanically Ventilated Patients With Sepsis: A Systematic Review and Meta-Analysis

**DOI:** 10.3389/fmed.2021.776882

**Published:** 2021-12-13

**Authors:** Caimu Wang, Qijiang Chen, Ping Wang, Weisheng Jin, Chao Zhong, Zisheng Ge, Kangmin Xu

**Affiliations:** General Intensive Care Unit, Ninghai First Hospital, Ningbo, China

**Keywords:** sepsis, anesthesiology, meta–analysis, dexmedetomidine, mechanical ventilation, sedation

## Abstract

**Purpose:** Dexmedetomidine has been shown to improve clinical outcomes in critically ill patients. However, its effect on septic patients remains controversial. Therefore, the purpose of this meta-analysis was to assess the effect of dexmedetomidine as a sedative agent for mechanically ventilated patients with sepsis.

**Methods:** We searched PubMed, Embase, Scopus, and Cochrane Library from inception through May 2021 for randomized controlled trials that enrolled mechanically ventilated, adult septic patients comparing dexmedetomidine with other sedatives or placebo.

**Results:** A total of nine studies involving 1,134 patients were included in our meta-analysis. The overall mortality (RR 0.97, 95%CI 0.82 to 1.13, *P* = *0.6*7, I^2^ = 25%), length of intensive care unit stay (MD −1.12, 95%CI −2.89 to 0.64, *P* = *0.2*1, I^2^ = 71%), incidence of delirium (RR 0.95, 95%CI 0.72 to 1.25, *P* = *0.7*0, I^2^ = 0%), and delirium free days (MD 1.76, 95%CI –0.94 to 4.47, *P* = 0.20, I^2^ = 80%) were not significantly different between dexmedetomidine and other sedative agents. Alternatively, the use of dexmedetomidine was associated with a significant reduction in the duration of mechanical ventilation (MD –0.53, 95%CI −0.85 to −0.21, *P* = *0.0*01, I^2^ = 0%) and inflammatory response (TNF-α: MD −5.27, 95%CI −7.99 to −2.54, *P*<*0.0*01, I^2^ = 0%; IL-1β: MD −1.25, 95%CI −1.91 to –0.59, *P*<*0.0*01, I^2^ = 0%).

**Conclusions:** For patients with sepsis, the use of dexmedetomidine as compared with other sedative agents does not affect all-cause mortality, length of intensive care unit stay, the incidence of delirium, and delirium-free days. But the dexmedetomidine was associated with the reduced duration of mechanical ventilation and inflammatory response.

## Introduction

Sepsis, defined as a life-threatening organ dysfunction due to a dysregulated immune response to infection, affects millions of patients per year and carries a high risk of mortality, becoming a major global health problem (1, 2). According to the Global Burden of Diseases Study, in 2017, an estimated 48.9 million incident cases of sepsis were reported worldwide with nearly 11.0 million patients dying, accounting for 19.7% of all global deaths (3).On the other hand, sepsis is often complicated with acute respiratory distress syndrome that requires mechanical ventilatory support (4, 5), research showed that more than 20% of septic patients needed invasive ventilation in the USA (6).

Sedation is an integral component of care for mechanically ventilated patients to reduce the anxiety and stress associated with tracheal intubation and other invasive interventions (7, 8). However, the appropriate choice of a preferred sedative agent for patients with sepsis remains controversial. For decades, γ-aminobutyric acid (GABA) receptor agonists (such as propofol and midazolam) were widely used as sedative drugs in the intensive care units (ICU) (9–11). Recently, dexmedetomidine, a highly selective α-2 adrenergic receptor agonist, is a unique alternative sedative compared with GABA receptor agonists considering its analgesic properties with wider safety margin due to the lack of suppressive effects on respiration (12). Using dexmedetomidine to induce sedation while preserving a degree of arousability for critically ill patients. Limited but increasing evidence suggests that dexmedetomidine has a promising future as a sedative agent in the ICU, its use resulted in a shorter duration of mechanical ventilation (MV) or ICU length of stay (13–17), a reduced incidence of coma or delirium (15, 17–19).

Recently, Hughes and coworkers conducted the MENDS II trial concerning the effect of dexmedetomidine vs. propofol on the short-term and long-term outcomes of mechanically ventilated adults with sepsis. In this pragmatic randomized controlled trial (RCT) involving more than 400 patients, there was no significant difference in the delirium or ventilator-free days, ICU length of stay, and 90-day mortality between the patients receiving dexmedetomidine or propofol. Therefore, the effects of dexmedetomidine in septic patients receiving MV remains controversial. We aimed to assess the effects of dexmedetomidine on clinical outcomes in mechanically ventilated patients with sepsis.

## Methods

Our study was conducted following the Preferred Reporting Items for Systematic Reviews and Meta-Analyses (PRISMA) statement (20) (Supplementary Material 1). The study protocol was registered in PROSPERO (CRD42019145061). A literature search was performed in PubMed, Embase, Scopus, and Cochrane Library for eligible RCTs in English from inception through May 2021. The search used broad search terms containing “sepsis,” “ventilation,” “dexmedetomidine,” and “randomized.” In addition, full details on our search terms and strategy detailed were recorded in Supplementary Material 2.

### Eligibility Criteria

Study inclusion criteria were as follows: (1). Population: adult (≥18 years old) patients with sepsis receiving mechanical ventilation and intravenous sedation; (2). Intervention: the use of IV dexmedetomidine regardless of dose, start time, and duration; (3). Comparison: the use of other IV sedative drugs or placebo regardless of dose, start time, and duration; (4). Outcomes: the primary outcome was overall mortality (including ICU, hospital, 28/30-day mortality). The secondary outcomes were duration of mechanical ventilation, ICU length of stay, and inflammatory responses (serum levels of inflammatory markers after 24 h). (5). Design: RCT.

The following studies would be excluded: (1). If the study evaluated obstetrical patients because sedation practices and mechanical ventilation strategies are different in the patient population (21); (2). Patients did not receive IV sedatives or mechanical ventilation; (3). Studies published only in abstract form.

### Data Extraction and Quality Assessment

Two authors independently retrieved relevant studies and extracted data from included studies. The characteristics of studies (first author, years of publication, study design, population, sedation goal, intervention, and control sedative agents, outcomes) were recorded in Table 1. Further information (study design, number of participants, sex ratio, mean age, inclusion, and exclusion criteria) was recorded in Supplementary Material 3.

**Table 1:** Characteristics of the included studies.

**First author, year**	**Design**	**Patients**	**Interventions**	**Sedation goals**	**Outcomes**
Hughes et al. (33)	Multicenter, double-blind	422 patients with sepsis	Intervention group: DEX for 0.15 to 1.5 μg/kg·h; Control group: propofol for 5 to 50 μg/kg·h	RASS score of −2 to 0	30-day mortality, 90-day mortality, delirium free days
Cioccari et al. (31)	Multicenter, open-label	83 patients with septic shock	Intervention group: DEX start at 1 μg/kg·h, followed at adjusted dose (maximunm at 1.5 μg/kg·h); Control group: propofol directed by the treating physician	RASS score of −2 to +1	ICU mortality, in-hospital mortality, 90-day mortality, duration of mechanical ventilation, ICU length of stay, incidence of delirium
Liu et al. (32)	Single-center, open-label	200 patients with septic shock	Intervention group: DEX start at a loading dose of 1 μg/kg·h, followed at 0.2 to 0.3 μg/kg·h; Control group: propofol start at a loading dose of 1 mg/kg, followed at 1 to 3 mg/kg·h	RASS score of −2 to 0	28-day mortality, duration of mechanical ventilation, ICU length of stay
Kawazoe et al. (30)	Multicenter, open-label	201 patients with sepsis	Intervention group: DEX start at start at 0.1 μg/kg·h then at 0.1–0.7 μg/kg·h; Control group: propofol at 0–3 mg/kg/h or midazolam at 0–0.15 mg/kg/h	RASS score of −2 to 0	28-day mortality, duration of mechanical ventilation, ICU length of stay, incidence of delirium
Guo et al. (29)	Single-center, open-label	45 patients with septic shock	Intervention group: DEX for 0.2 to 0.7 μg/kg·h; Control group 1: propofol; Control group 2: midazolam;	RASS score of −2 to −1	In-hospital mortality, duration of mechanical ventilation, ICU length of stay
Pandharipande et al. (28)	Multicenter, double-blind	63 patients with sepsis	Intervention group: DEX at a median rate of 0.74 μg/kg·h, max does at 1.5 μg/kg·h; Control group: lorazepam at a median rate of 3 mg/h, max does at 10 mg/h	RASS score of −2 to +1	28-day mortality, ICU length of stay, delirium free days
Tasdogan et al. (27)	Single-center, open-label	40 patients with sepsis	Intervention group: DEX at a loading does of 1 μg/kg for 10 min, followed by 0.2–2.5 μg/kg·h; Control group: propofpl at a loading does of 1 mg/kg·h for 15 min, followed by 1–3 mg/kg·h	RSS≤2	28-day mortality, duration of mechanical ventilation, ICU length of stay, levels of TNF-α and IL-1β
Memis et al. (27)	Single-center, open-label	40 patients with septic shock	Intervention group: DEX at a loading does of 1 μg/kg for 10min, followed by 0.2–2.5 μg/kg·h; Control group: propofpl at a loading does of 1 mg/kg·h for 15 min, followed by 1–3 mg/kg·h	RSS≤2	ICU mortality, ICU length of stay
Memis et al. (27)	Single-center, open-label	40 patients with sepsis	Intervention group: DEX at a loading does of 1 μg/kg for 10 min, followed by 0.2–2.5 μg/kg·h; Control group: midazolam at a loading does of 0.2 mg/kg·h for 10 min, followed by 0.1–0.5 mg/kg·h	RSS < 2	ICU mortality, levels of TNF-α and IL-1β

*DEX, dexmedetomidine; RASS, Richmond agitation sedation scale; RSS, Ramsay sedation score*.

Two authors independently assessed the methodological quality of the included studies by using the Cochrane risk of bias tool (22).

### Statistical Synthesis and Analysis

We combined the data from the included studies to estimate the pooled relative ratio (RR) with a 95% CI for the primary outcome, and the secondary outcomes were pooled as mean difference (MD) with 95%CI.

The heterogeneity between studies was tested by the Chi-squared test with significance set at a *P*-value of 0.1, and quantitatively by inconsistency (I^2^) statistics (23). Substantial heterogeneity was identified when I^2^ > 30% and we employed a random-effects model to perform the analysis, otherwise, a fixed-effects model would be used. In addition, we used the funnel plot and Egger's regression test to assess the publication bias (24).

A predefined subgroup analysis was stratified by population (sepsis or septic shock) and control drug (propofol or others) to investigate the potential source of heterogeneity. Furthermore, we performed a sensitivity analysis to explore the effect of individual studies by omitting each one at a time.

## Results

### Study Identification and Characteristics

**Figure 1:**
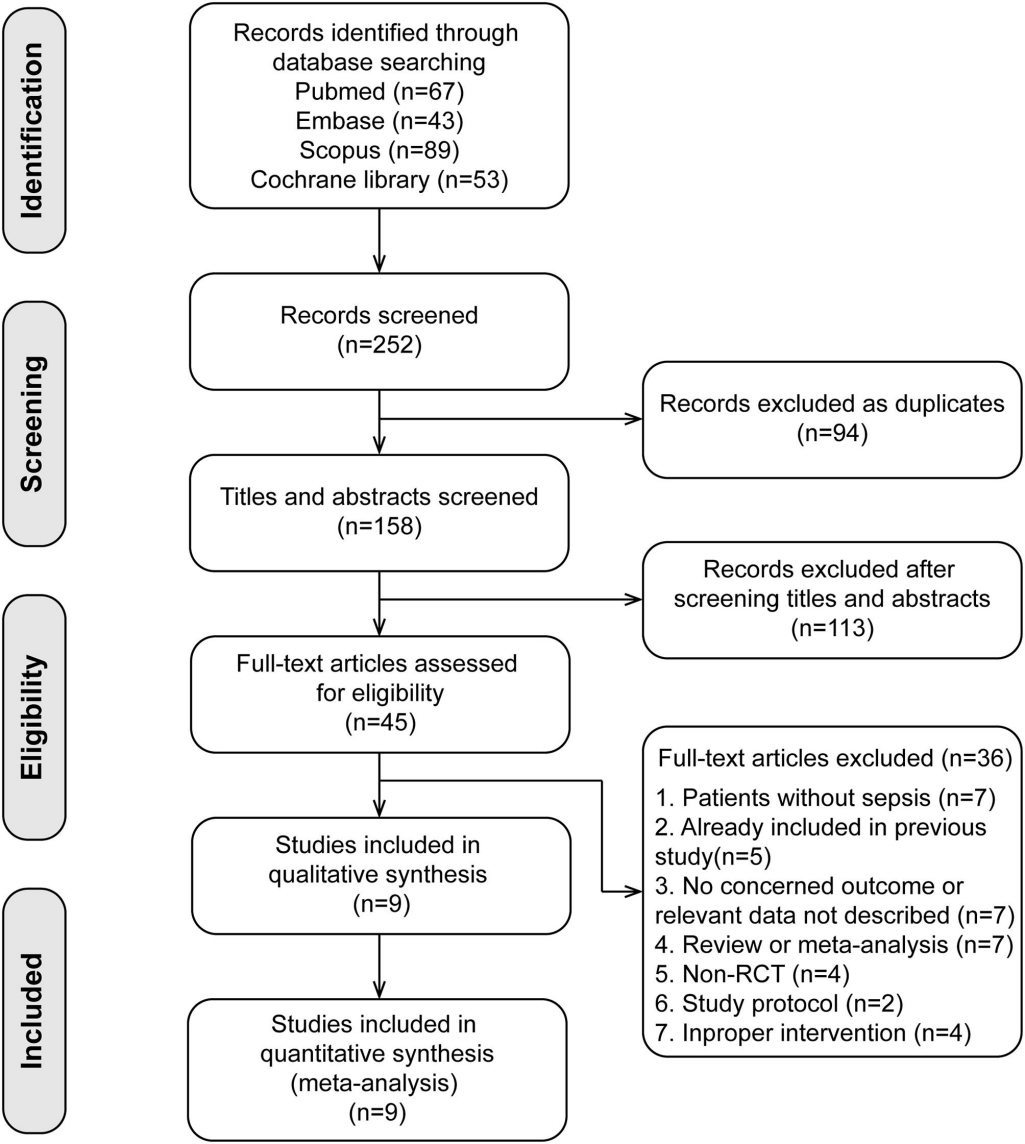
Flow chart of database search and study selection.

A total of nine studies (25–33) involving 1,134 patients were included (search process in Figure 1). Among the nine included studies, two studies were subgroup analyses of patients with sepsis from previous RCTs [Cioccari et al. (31) performed a subgroup analysis of the SPICE III trial (34), Pandharipande et al. (28) performed a subgroup analysis of the MENDS trial (35)]. Table 1 shows the characteristics of the included studies. Five trials used dexmedetomidine in patients with sepsis (25, 27, 28, 30, 33), and four in patients with septic shock (26, 29, 31, 32). Different control drugs were also identified: propofol in six arms (26, 27, 29, 31–33), midazolam in two arms (25, 29), lorazepam in one arm (28), and propofol plus midazolam in one arm (30). Five studies (25–27, 31, 32) used a loading dose of dexmedetomidine at 1 μg/kg·h, the other four studies (28–30, 33) used infusion dose of dexmedetomidine between 0.1 to 1.5 μg/kg·h. The target sedation goals were Richmond Agitation Sedation Scale of −2 to 0 in three trials (30, 32, 33), −2 to 1 in two trials (28, 31), −2 to −1 in one trial (29), and Ramsay Sedation Score <2 in three trials (25–27).

Other detailed information of included studies (e.g., study design, sex ratio, age, APACHE II and SOFA score at trial enrollment, definitions of sepsis or septic shock) were reported in Supplementary Material 3.

### Quality Assessment

**Figure 2:**
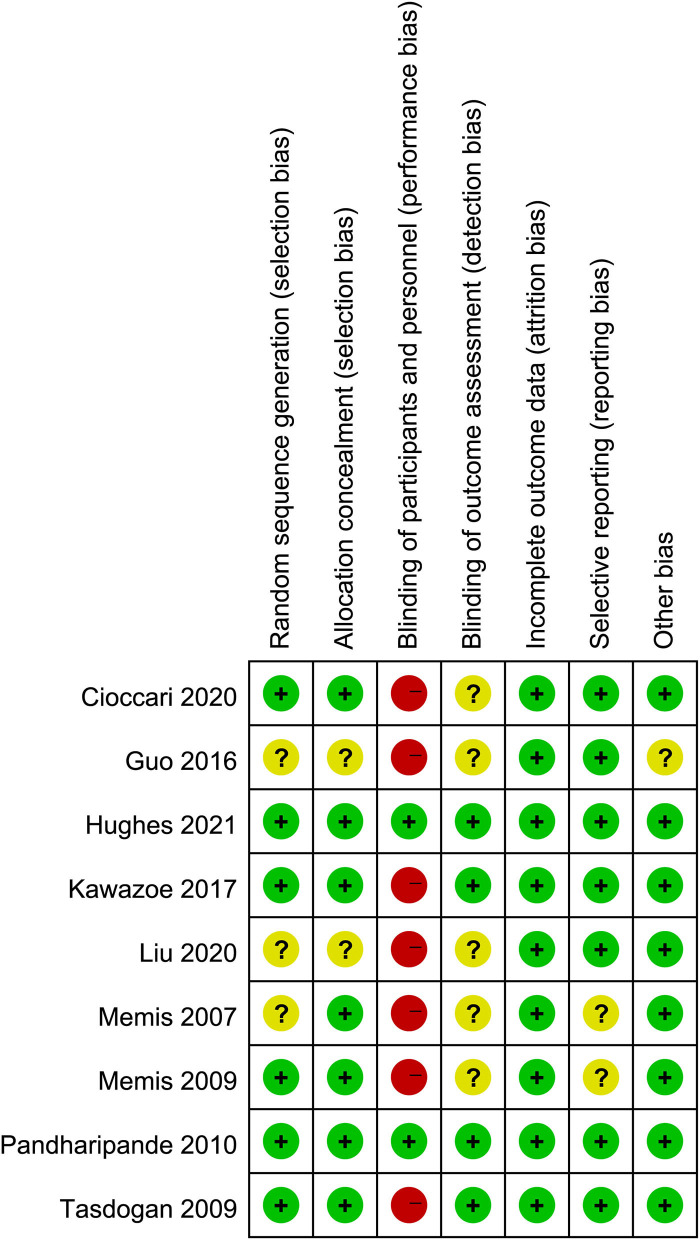
Assessment of quality by the Cochrane risk of bias tool. Red denotes high risk, yellow unclear risk and green low risk.

The quality assessment by the Cochrane risk of bias tool was summarized in Figure 2. Seven studies (25–27, 29–32) had a high risk of bias because they were open-label trials. Three studies (25, 29, 32) did not report the details of random sequence generation and allocation concealment. The blinding method for outcome assessment was not reported in five studies (25, 26, 29, 31, 32), which would either underestimate or overestimate the size of the effect. Moreover, in the trial by Guo et al. (29), the doses of control sedative drugs were not reported. The details for quality assessment and reason for judgment were recorded in Supplementary Material 3.

We used the funnel plot and Egger's test to assess the publication bias (Supplementary Material 4), the results showed there was a potential risk of publication bias for the overall mortality and length of ICU stay (Egger's test, *P* < 0.01). Thus, we performed an additional analysis by using the trim and fill method. The imputed studies produced a symmetrical funnel plot and the analysis after imputing continued to show no association between the use of dexmedetomidine and overall mortality (RR 1.05, 95%CI 0.91 to 1.22 *P* = *0.6*7, I^2^ = 32%), but a shortened length of ICU stay was observed (MD −2.42, 95%CI −4.15 to –0.68, *P*<*0.0*1, I^2^ = 75%).

### Primary Outcome

**Figure 3:**
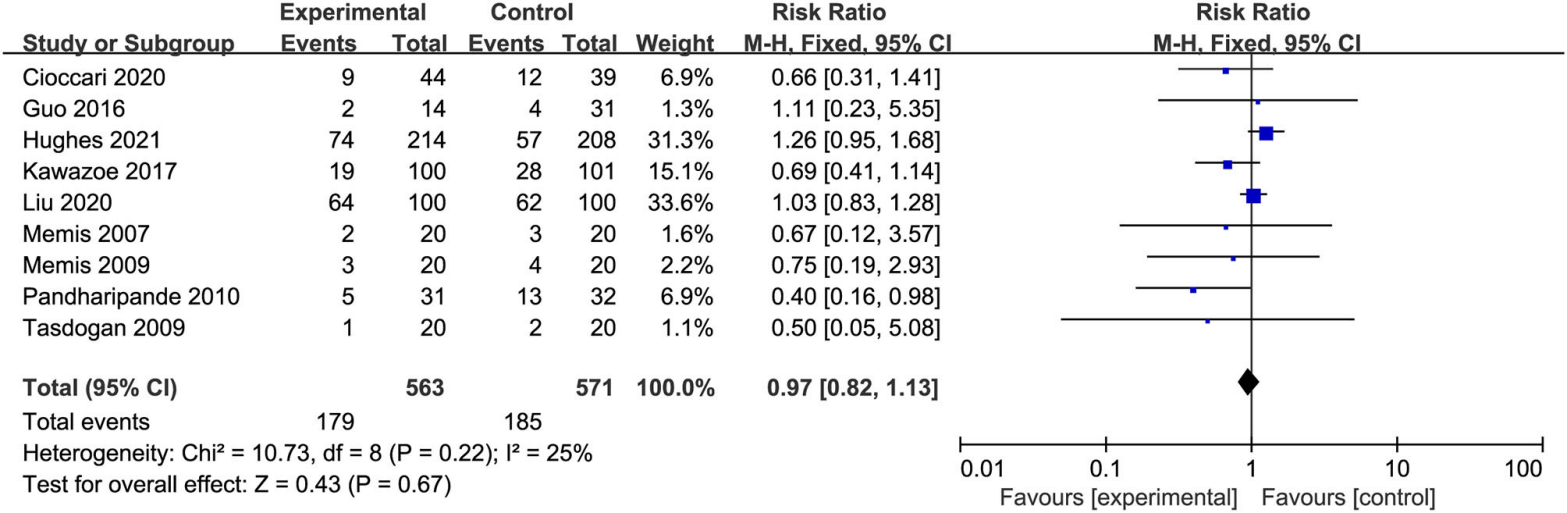
Forest plot comparing the effect of DEX on mortality.

All studies involving 1,134 patients reported mortality (two studies reported the ICU mortality, four studies reported the 28/30-day mortality, one study reported the in-hospital mortality, one study reported the 30-day and 90-day mortality, one study reported the ICU, in-hospital, and 90-day mortality). The risk for overall mortality was similar between dexmedetomidine and control regimens without significant heterogeneity (RR 0.97; 95%CI 0.82 to 1.13, *P* = *0.6*7, I^2^ = 25%; Table 2, Figure 3). Similarly, the use of dexmedetomidine had no significant effect on the ICU mortality, 28/30-day mortality, in-hospital mortality, and 90-day mortality (Table 2, Supplementary Material 5).

**Table 2:** Primary and secondary outcomes of this meta-analysis.

**Outcome**	**N**	**Result**
Overall mortality	9	RR 0.97, 95%CI 0.82 to 1.13, *P* = 0.67, I^2^ = 25%
ICU mortality	3	RR 0.60, 95%CI 0.30 to 1.21, *P* = 0.15, I^2^ = 0%
28/30-day mortality	5	RR 1.00, 95%CI 0.84 to 1.18, *P* = 0.98, I^2^ = 56%
In-hospital mortality	2	RR 0.74, 95%CI 0.38 to 1.45, *P* = 0.37, I^2^ = 0%
90-day mortality	2	RR 0.94, 95%CI 0.75 to 1.18, *P* = 0.67, I^2^ = 29%
Length of ICU stay	8	MD −1.12, 95%CI −2.89 to 0.64, *P* = 0.21, I^2^ = 71%
Duration of MV	6	MD −0.53, 95%CI −0.85 to −0.21, *P* = 0.001, I^2^ = 0%
Inflammatory response		
TNF-α	2	MD −5.27, 95%CI −7.99 to −2.54, *P* < 0.001, I^2^ = 0%
IL-1β	2	MD −1.25, 95%CI −1.91 to −0.59, *P* < 0.001, I^2^ = 0%
Incidence of delirium	2	RR 0.95, 95%CI 0.72 to 1.25, *P* = 0.70, I^2^ = 0%
Delirium free days	2	MD 1.76, 95%CI −0.94 to 4.47, *P* = 0.20, I^2^ = 80%

*N, number of studies; ICU, intensive care unit; RR, risk ratio; CI, confidence interval; MV, mechanical ventilation; MD, mean difference; TNF-α, tumor necrosis factor-α; IL-1β, interleukin-1β*.

**Figure 4:**
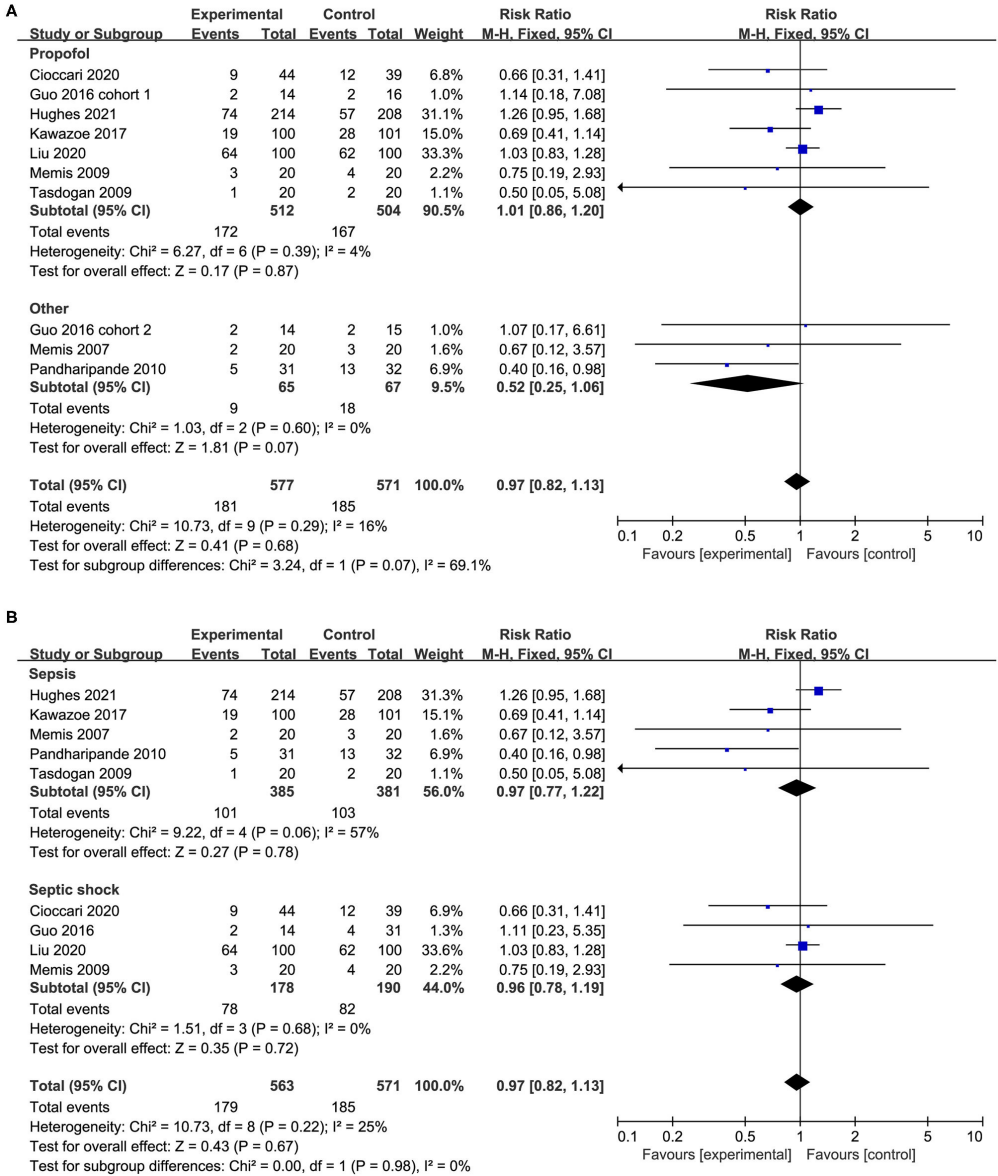
Subgroup analysis for mortality **(A)** propofol vs. other sedation, **(B)** sepsis vs. septic shock.

We performed predefined subgroup analyses stratified by population (sepsis or septic shock) and control drug (propofol or others). Compared with propofol, the use of dexmedetomidine does not affect the overall mortality (RR 1.01, 95%CI 0.86 to 1.20, *P* = *0.8*7, I^2^ = 4%; Figure 4A). However, a trend toward the reduction of all-cause mortality by dexmedetomidine when compared with other sedations (RR 052, 95%CI 0.25 to 1.06, *P* = *0.0*7, I^2^ = 0%; Figure 4A) was observed, although it was not statistically significant. The use of dexmedetomidine had no different effect on mortality for patients with sepsis or septic shock (Figure 4B). Moreover, the sensitivity analysis by removing each trial showed similar results to the overall analysis, indicating good robustness (Supplementary Material 5).

### Secondary Outcomes

**Figure 5:**
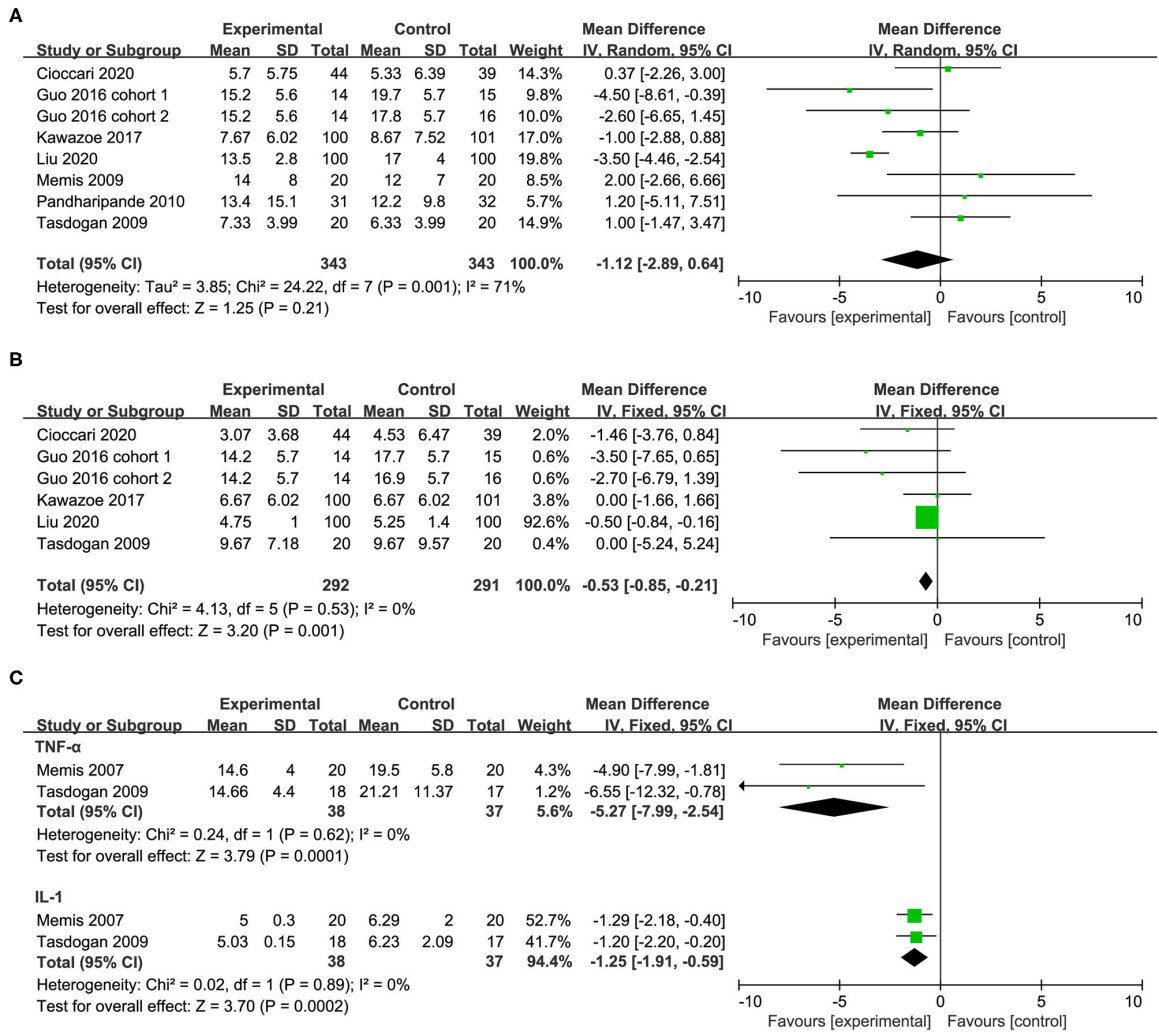
Forest plot comparing the effect of DEX on **(A)** length of ICU stay, **(B)** duration of MV, **(C)** inflammatory response.

A total of seven studies with eight cohorts reported the length of ICU stay and there was no significant difference between patients who received dexmedetomidine and other sedative drugs (MD −1.12, 95%CI −2.89 to 0.64, *P* = *0.2*1, I^2^ = 71%; Table 2, Figure 5A). The overall analysis from five studies (six cohorts) showed that the use of dexmedetomidine was associated with a slight reduction in the duration of MV (MD −0.53, 95%CI −0.85 to −0.21, *P* = *0.0*01, I^2^ = 0%; Table 2, Figure 5B). However, since the significant heterogeneity, this result should be interpreted prudently.

In the subgroup analysis, compared with propofol, the use of dexmedetomidine was associated with the shortened duration of MV, but no significant difference in the length of ICU stay. Furthermore, the population (sepsis or septic shock) has no significant effect on the length of ICU stay. The positive effect of dexmedetomidine in reducing the duration of MV became not significant in the sepsis subgroup (Table 2, Supplementary Material 5). The sensitivity analysis by removing each trial showed no significant difference in the length of ICU stay, indicating good robustness. The positive effect of dexmedetomidine in reducing the duration of MV became not significant after omitting the study by Liu et al. (32) (Supplementary Material 5).

Two studies reported the serum levels of inflammatory markers including the tumor necrosis factor α (TNF-α) and interleukin 1β (IL-1β) after 24 h of treatment. In the group of patients receiving dexmedetomidine, the serum levels of TNF-α and IL-1β after 24 h of treatment were significantly lower than that in the control group (TNF-α: MD −5.27, 95%CI −7.99 to −2.54, *P*<*0.0*01, I^2^ = 0%; IL-1β: MD −1.25, 95%CI −1.91 to −0.59, *P*<*0.0*01, I^2^ = 0%; Table 2, Figure 5C).

**Figure 6:**
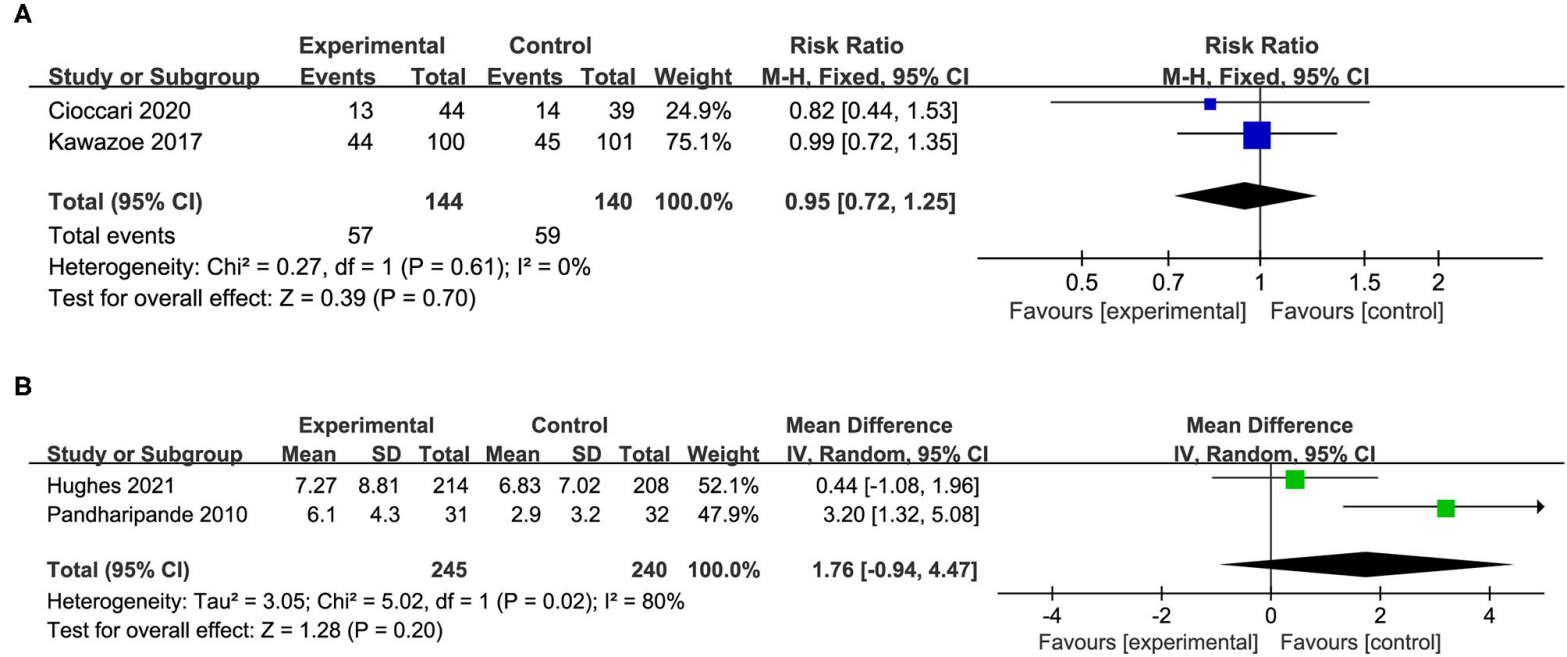
Forest plot comparing the effect of DEX on **(A)** incidence of delirium, **(B)** delirium free days.

In addition, four studies reported the incidence of delirium or the delirium-free days. The results indicated that the use of dexmedetomidine had no significant effect on the incidence of delirium (RR 0.95, 95%CI 0.72 to 1.25, *P* = *0.7*0, I^2^ = 0%; Table 2, Figure 6A) and delirium free days (MD 1.76, 95%CI −0.94 to 4.47, *P* = *0.2*0, I^2^ = 80%; Table 2, Figure 6B).

Furthermore, there were no differences in the use of vasopressor, the incidence of hypotensive, and mean arterial pressure during the study period between the dexmedetomidine and control groups (Supplementary Material 6).

## Discussion

In this meta-analysis, we systematically and comprehensively reviewed nine studies with 1,134 patients to evaluate the effect of dexmedetomidine for mechanically ventilated patients with sepsis. Overall, compared with other sedation, the use of dexmedetomidine does not affect the overall mortality, length of ICU stay, incidence of delirium, or the delirium-free days. We found that patients with sepsis receiving dexmedetomidine had a shortened duration of MV, limited evidence suggested that the use of dexmedetomidine was associated with a lower level of TNF-α and IL-1β. Moreover, we found survival benefits in comparison with other sedations including lorazepam and midazolam, but not with propofol.

There are three previous systematic reviews and meta-analyses (36–38) comparing the effect of dexmedetomidine vs. other sedative agents on clinical outcomes of patients with sepsis. The results of previous studies showed that compared with other sedatives, the use of dexmedetomidine had beneficial effects on 28-day mortality, but there were no significant differences in the length of ICU stay and duration of MV (36–38). Our meta-analysis indicated that the use of dexmedetomidine was associated with the shortened duration of MV for mechanically ventilated patients with sepsis, but no significant differences in the overall mortality. These differences may result from several newly published RCTs. In contrast to previous meta-analyses, our research updated the findings by including recently published RCTs (31–33), especially the MENDS II trial. In this pragmatic RCT involving 400 patients from 13 medical centers, there are many important methodologic advances including a higher degree of trial drug allocation concealment and blinding, better separation between groups concerning sedative exposure, and stricter adherence to light sedation approaches (33). The increased trials and number of patients could provide more robust results. Moreover, in the subgroup analyses, we found survival benefits in comparison with lorazepam, not with propofol.

Dexmedetomidine could promote macrophage phagocytosis and bactericidal killing further enhancing the mucosal immunity and bacterial clearance, which are of great importance for patients with sepsis and septic shock. Previous research has shown that dexmedetomidine had potential anti-inflammatory effects in both animal and human studies (39). Results of our meta-analysis also suggest that after 24 h of receiving dexmedetomidine, the levels of TNF-α and IL-1β were significantly lower than the control group. This anti-inflammatory effect of dexmedetomidine can suppress the exaggerated production of inflammatory cytokines in septic shock (4, 40, 41).

The strengths of our study include the comprehensive and up-to-date search strategies, specific and targeted inclusion criteria, comprehensive and rigorous analytical methods. Three studies included in our meta-analysis are recently published RCTs (31–33) with large populations. The predefined subgroup analysis found survival benefits of dexmedetomidine in comparison with other sedations including lorazepam and midazolam.

However, our meta-analysis also had several limitations. First of all, the main limitation is the limited number of included studies and the small sample size. Six of the included trials are typically characterized as small studies because the sample size was smaller than 100 patients, which may lead to small study effect bias (42). Second, the diagnostic criteria for sepsis or septic shock, the dose of the sedative drug, and target sedation goals were varied among included studies. These factors may cause clinical heterogeneity. Third, the follow-up duration in most included studies was relatively short, only two studies reported 90-day mortality as the long-term outcome. More RCTs with long follow-ups were necessary to demonstrate the effects of dexmedetomidine on long-term outcomes. Last but not the least, in some included studies, the dexmedetomidine was combined with other sedative agents including opioids or benzodiazepines in the intervention group for sedation. Therefore, the actual efficacy of single administration with dexmedetomidine for patients with sepsis requires further validation.

## Conclusion

In conclusion, our meta-analysis suggests that for patients with sepsis or septic shock, the use of dexmedetomidine has no effect on all-cause mortality and length of ICU stay, but may offer advantages in terms of reducing the duration of mechanical ventilation and inflammatory response. However, considering the significant heterogeneity and the limited number of included studies with small sample sizes. Well-designed, multicenter RCTs with a large sample size are needed to further evaluate the effect of dexmedetomidine on short-term and long-term clinical outcomes for patients with sepsis and to compare its effects with other sedative agents.

## Data Availability Statement

The original contributions presented in the study are included in the article/Supplementary Material, further inquiries can be directed to the corresponding author.

## Author Contributions

CW contributed to the acquisition and analysis of the data, the initial draft writing of this paper, and the final approval of the version to be published. QC, PW, and WJ contributed to the collection and interpretation of data. CZ, ZG, and KX contributed to the concept of the review and the revision of this paper. All authors contributed to the article and approved the submitted version.

## Funding

This work was supported in part by grants from the Medical and Health Research Program of Zhejiang Province (No. 2020KY907). The sponsor of this study had no role in study design, data collection, data analysis, data interpretation, or writing of the report.

## Conflict of Interest

The authors declare that the research was conducted in the absence of any commercial or financial relationships that could be construed as a potential conflict of interest.

## Publisher's Note

All claims expressed in this article are solely those of the authors and do not necessarily represent those of their affiliated organizations, or those of the publisher, the editors and the reviewers. Any product that may be evaluated in this article, or claim that may be made by its manufacturer, is not guaranteed or endorsed by the publisher.
