# Supplementary material for: The Effect of Dexmedetomidine as a Sedative Agent for Mechanically Ventilated Patients With Sepsis: A Systematic Review and Meta-Analysis
Source: Front Med (Lausanne). 2021 Dec 13;8:776882. doi: 10.3389/fmed.2021.776882 (PMC8711777; doi:10.3389/fmed.2021.776882)
Supplement: Supplementary file 1 [file Data_Sheet_1.docx]

**Supplementary Material 1: Searching strategies**

**Pubmed 67**

#1 sepsis [MeSH Terms] OR sepsis [Title/Abstract] OR septic [Title/Abstract]

#2 dexmedetomidine [Title/Abstract] OR dexmedetomidine [MeSH Terms]

#3 randomized controlled trial [MeSH Terms] OR random* [Title/Abstract]

#1 AND #2 AND #3

**Embase 43**

#1 ‘sepsis’:ti,ab,kw OR ‘septic’:ti,ab,kw OR ' sepsis '/exp OR ' septic '/exp

#2 dexmedetomidine:ti,ab,kw OR ' dexmedetomidine '/exp

#3 'randomized controlled trial'/de OR 'randomized controlled trial'/exp

#1 AND #2 AND #3

**Scopus 89**

#1 TITLE-ABS-KEY (sepsis) OR TITLE-ABS-KEY (septic)

#2 TITLE-ABS-KEY (dexmedetomidine)

#3 TITLE-ABS-KEY (randomized) OR TITLE-ABS-KEY (random) OR TITLE-ABS-KEY (randomised)

#1 AND #2 AND #3

**Cochrane Library 53**

#1 (sepsis):ti,ab,kw OR (septic):ti,ab,kw

#2 (dexmedetomidine):ti,ab,kw

#3 (randomized):ti,ab,kw OR (randomised):ti,ab,kw OR (random):ti,ab,kw

#1 AND #2 AND #3
