# Supplementary material for: The Effect of Dexmedetomidine as a Sedative Agent for Mechanically Ventilated Patients With Sepsis: A Systematic Review and Meta-Analysis
Source: Front Med (Lausanne). 2021 Dec 13;8:776882. doi: 10.3389/fmed.2021.776882 (PMC8711777; doi:10.3389/fmed.2021.776882)
Supplement: Supplementary file 2 [file Data_Sheet_2.docx]

**Supplementary Material 2: Detailed information of included studies**

| First author (Year) | Hughes 2021 |
| --- | --- |
| Study type | Multicenter, double-blind, randomized, controlled trial |
| Baseline characteristics  (DEX/Control) Number Male% Age  APACHE II  SOFA | 214/208  57/58  59/60 (median)  27/27 (median)  10/10 (median) |
| Inclusion criteria | Adults who were sequentially admitted to a medical or surgical ICU, had suspected or known infection, and were treated with continuous sedation for invasive mechanical ventilation |
| Bias  Selection bias      Performance bias    Detection bias  Attrition bias  Reporting bias  Other bias | Low (Patients were randomly assigned to receive dexmedetomidine or propofol in a 1:1 ratio using computer-generated permuted blocks stratified by enrollment site and age)  Low (Researchers, clinicians, patients were unaware of the group assignments)  Low  Low  Low  Low |

| First author (Year) | Cioccari 2020 |
| --- | --- |
| Study type | Multicenter, open-label, randomized, controlled trial |
| Baseline characteristics  (DEX/Control) Number Male% Age  APACHE II  SOFA | 44/39  66/72  68/63 (mean)  25/25 (mean)  NR |
| Inclusion criteria | Patients receiving mechanical ventilation beyond the next full calendar day, receiving sedatives for safety and comfort, had documented or strong suspicion of infection with at least 2 SIRS criteria, and receiving administration of vasopressors or inotropes prior to randomization and for a cumulative duration of ≥ 4 h to maintain blood pressure targets set by the treating clinician |
| Bias  Selection bias  Performance bias  Detection bias  Attrition bias  Reporting bias  Other bias | Low (Computer-based center randomization program, block randomization with variable block size)  High (All sedatives were administered in an open-label manner)  Unclear (Lack of information)  Low  Low  Low |

| First author (Year) | Liu 2020 |
| --- | --- |
| Study type | Single-center, open-label, randomized, controlled trial |
| Baseline characteristics  (DEX/Control) Number Male% Age  APACHE II  SOFA | 100/100  57/58  57/54 (median)  29/29 (median)  10/11 (median) |
| Inclusion criteria | Patients with septic shock undergoing mechanical ventilation. Septic shock was defined as sepsis with hypotension unresponsive to fluid resuscitation and further requiring vasopressors to maintain blood pressure on admission day |
| Bias  Selection bias  Performance bias  Detection bias  Attrition bias  Reporting bias  Other bias | Unclear (Lack of information)  High (All sedatives were administered in an open-label manner)  Unclear (Lack of information)  Low  Low  Low |

| First author (Year) | Kawazoe 2017 |
| --- | --- |
| Study type | Multicenter, open-label, randomized, controlled trial |
| Baseline characteristics  (DEX/Control) Number Male% Age  APACHE II  SOFA | 100/101  63/63  68/69 (median)  27/27 (median)  10/10 (median) |
| Inclusion criteria | Patients aged 20 years or older, had sepsis, needed mechanical ventilation for at least 24 hours, sepsis was defined as systemic inflammatory response syndrome due to infection. |
| Bias  Selection bias    Performance bias  Detection bias  Attrition bias  Reporting bias  Other bias | Low (Permuted block randomization stratified by study center, presence of emergency surgery, chronic obstructive pulmonary disease, and soft tissue infection)  High (All sedatives were administered in an open-label manner)  Low  Low  Low  Low |

| First author (Year) | Guo 2016 |
| --- | --- |
| Study type | Single-center, open-label, randomized, controlled trial |
| Baseline characteristics  (DEX/Control) Number Male% Age  APACHE II  SOFA | 14/15/16  71/69/67  55/58/62 (mean)  24/23/21 (mean)  NR |
| Inclusion criteria | Patients had septic shock, needed mechanical ventilation at ICU admission, sepsis was defined according to the surviving sepsis campaign guidelines |
| Bias  Selection bias  Performance bias  Detection bias  Attrition bias  Reporting bias  Other bias | Unclear (Lack of information)  High (All sedatives were administered in an open-label manner)  Unclear (Lack of information)  Low  Low  Unclear (The dose of sedatives was unclear) |

| First author (Year) | Pandharipande 2010 |
| --- | --- |
| Study type | Multi-center, double-blinded, randomized controlled trial |
| Baseline characteristics  (DEX/Control) Number Male% Age  APACHE II  SOFA | 31/32  58/41  60/58 (median)  30/29 (median)  10/9 (median) |
| Inclusion criteria | Adult patients with sepsis requiring mechanical ventilation for longer than 24 hours |
| Bias  Selection bias  Performance bias    Detection bias  Attrition bias  Reporting bias  Other bias | Low (Computer-generated, permuted block randomization)  Low (Patients and all study personnel were blinded to study drug assignment)  Low  Low  Low  Low |

| First author (Year) | Tasdogan 2009 |
| --- | --- |
| Study type | Single-center, open-label, randomized, controlled trial |
| Baseline characteristics  (DEX/Control) Number Male% Age  APACHE II  SOFA | 20/20  70/55  58/50 (median)  19/18 (mean)  4/4 (mean) |
| Inclusion criteria | Adult septic patients admitted to the ICU after ileus surgery and who were expected to require postoperative sedation and ventilation |
| Bias  Selection bias  Performance bias  Detection bias  Attrition bias  Reporting bias  Other bias | Low (Computer generated table, sealed envelopes)  High (No blinding in the study)  Unclear (Lack of information)  Low  Low  Low |

| First author (Year) | Memis 2009 |
| --- | --- |
| Study type | Single-center, open-label, randomized, controlled trial |
| Baseline characteristics  (DEX/Control) Number Male% Age  APACHE II  SOFA | 20/20  70/65  60/54 (median)  22/20 (mean)  5/4 (mean) |
| Inclusion criteria | Adult patients with early septic shock, as outlined in the 2001 SCCM/ESICM/ACCP/ATS/SIS |
| Bias  Selection bias  Performance bias  Detection bias  Attrition bias  Reporting bias  Other bias | Low (Computer generated table, sealed envelopes)  High (No blinding in the study)  Unclear (Lack of information)  Low  Unclear (The study protocol was not obtainable)  Low |

| First author (Year) | Memis 2007 |
| --- | --- |
| Study type | Single-center, open-label, randomized, controlled trial |
| Baseline characteristics  (DEX/Control) Number Male% Age  APACHE II  SOFA | 20/20  NR  NR  NR  NR |
| Inclusion criteria | Critically ill patients with bacteriologically documented infections were included in the study if they met at least two of the criteria of sepsis, defined by the ACCP and SCCM |
| Bias  Selection bias  Performance bias  Detection bias  Attrition bias  Reporting bias  Other bias | Low (Computer generated table, sealed envelopes)  High (No blinding in the study)  Unclear (Lack of information)  Low  Unclear (The study protocol was not obtainable)  Low |
