# Supplementary material for: The Effect of Dexmedetomidine as a Sedative Agent for Mechanically Ventilated Patients With Sepsis: A Systematic Review and Meta-Analysis
Source: Front Med (Lausanne). 2021 Dec 13;8:776882. doi: 10.3389/fmed.2021.776882 (PMC8711777; doi:10.3389/fmed.2021.776882)
Supplement: Supplementary file 3 [file Data_Sheet_3.docx]

**Supplementary Material 3:** Publication bias assessed by funnel plot and Egger’s regression test


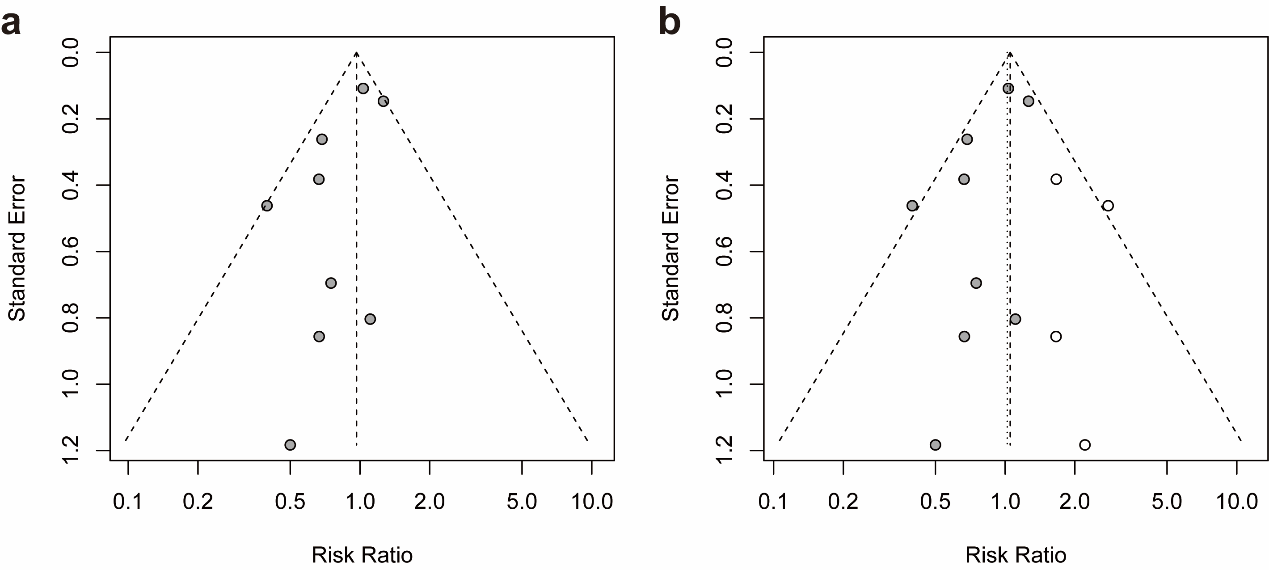


**Figure 1:** (a) funnel plot for overall mortality, Egger’s test (P=0.0776); (b) symmetrical funnel plot after trim and fill method for overall mortality (RR 1.05, 95%CI 0.91 to 1.22, I2=32%, P=0.67).


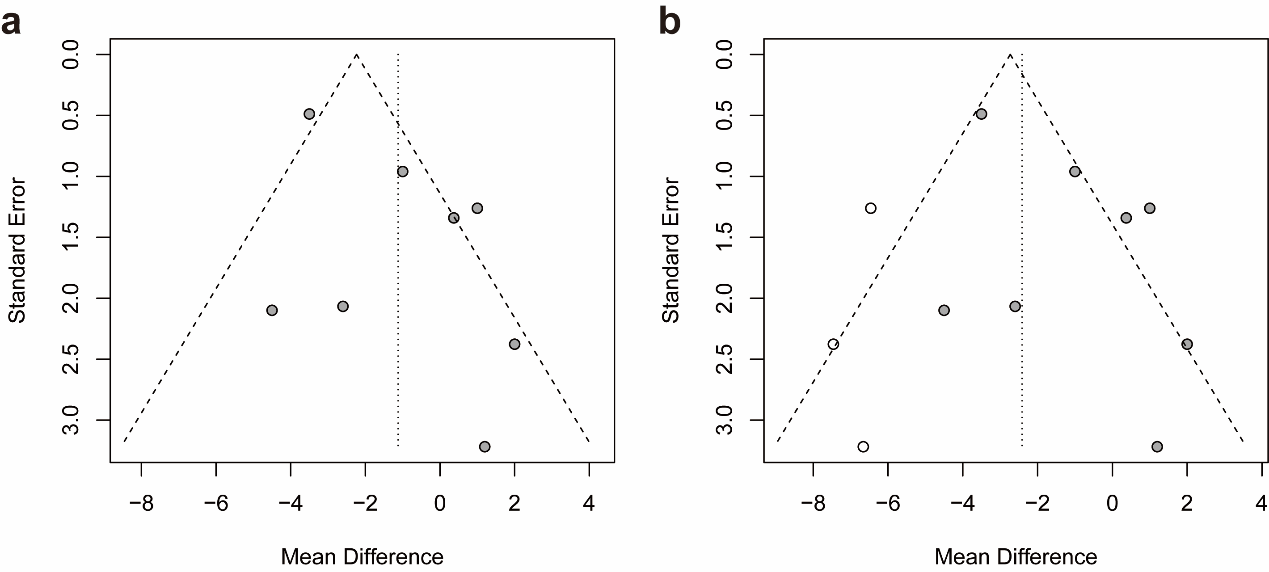


**Figure 2:** (a) funnel plot for length of ICU stay, Egger’s test (P=0.0991); (b) symmetrical funnel plot after trim and fill method for length of ICU stay (MD -2.42, 95%CI -4.15 to -0.68, I2=75%, P<0.01).


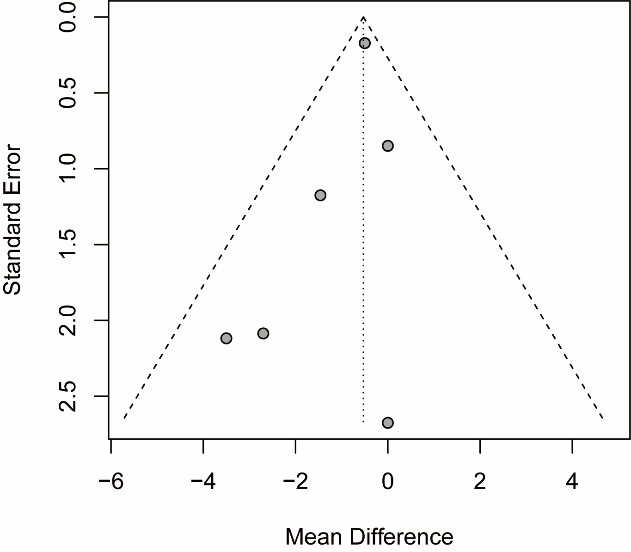


**Figure 3:** Funnel plot for length of duration of MV, Egger’s test (P=0.2361)
