# Supplementary material for: The Effect of Dexmedetomidine as a Sedative Agent for Mechanically Ventilated Patients With Sepsis: A Systematic Review and Meta-Analysis
Source: Front Med (Lausanne). 2021 Dec 13;8:776882. doi: 10.3389/fmed.2021.776882 (PMC8711777; doi:10.3389/fmed.2021.776882)
Supplement: Supplementary file 4 [file Data_Sheet_4.docx]

**Supplementary File 4: Forest plot for the subgroup and sensitivity analyses**


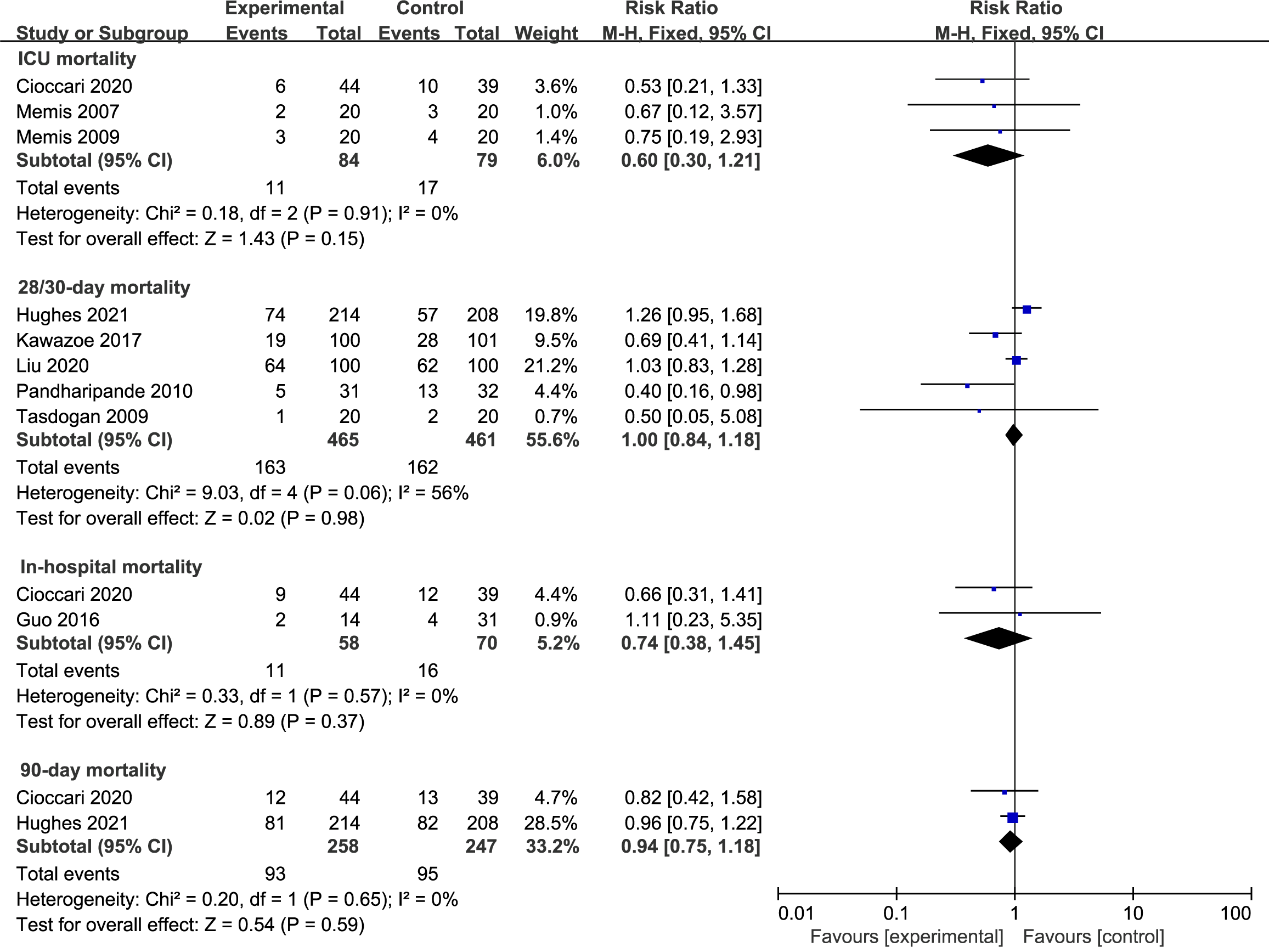


Figure 1: Forest plot for the ICU mortality, 28/30-day mortality, in-hospital mortality, 90-day mortality


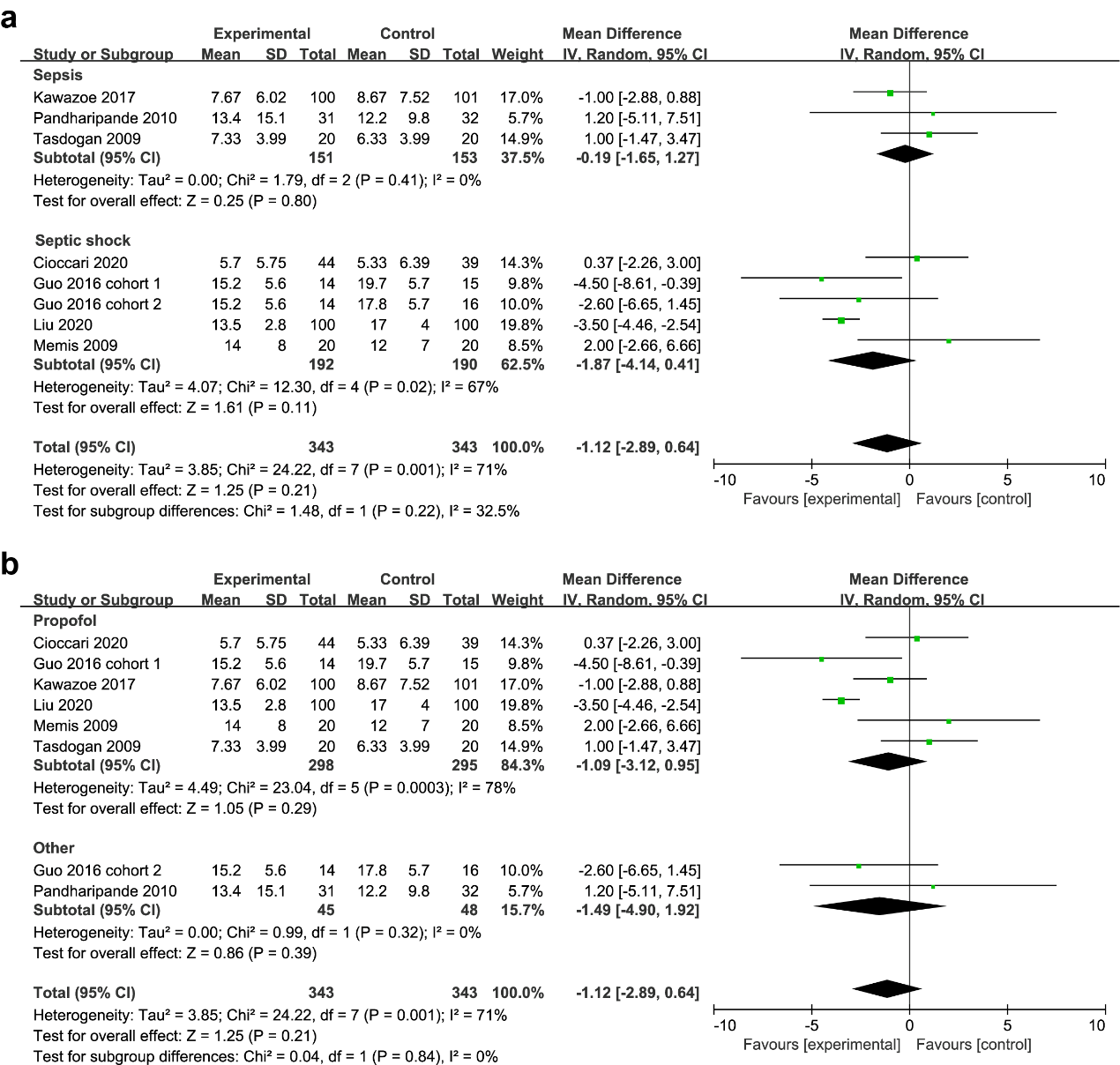


Figure 2: Subgroup analysis of the length of ICU stay; (a) according to population; (b) according to control drug


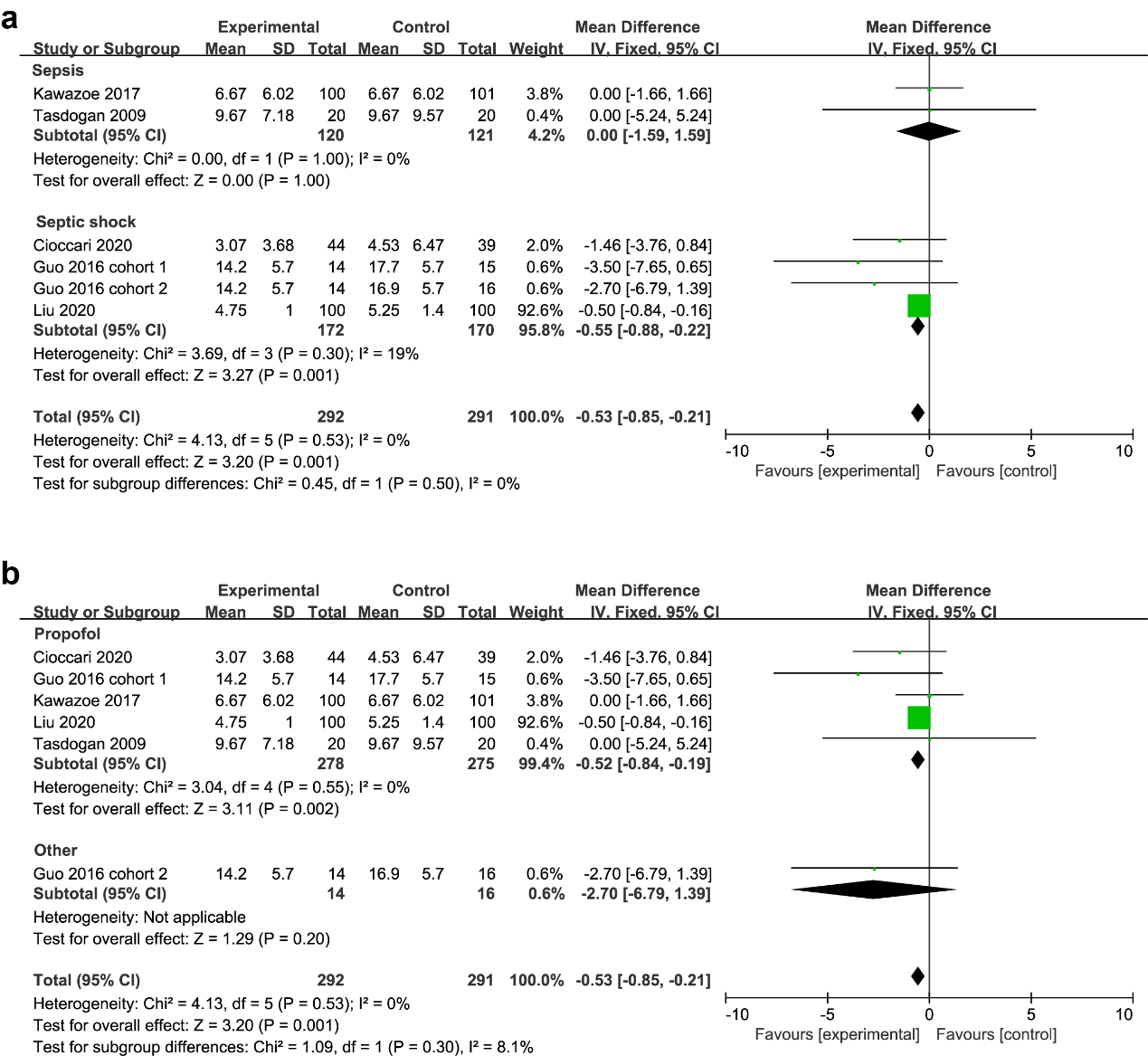


Figure 3: Subgroup analysis of the duration of MV; (a) according to population; (b) according to control drug


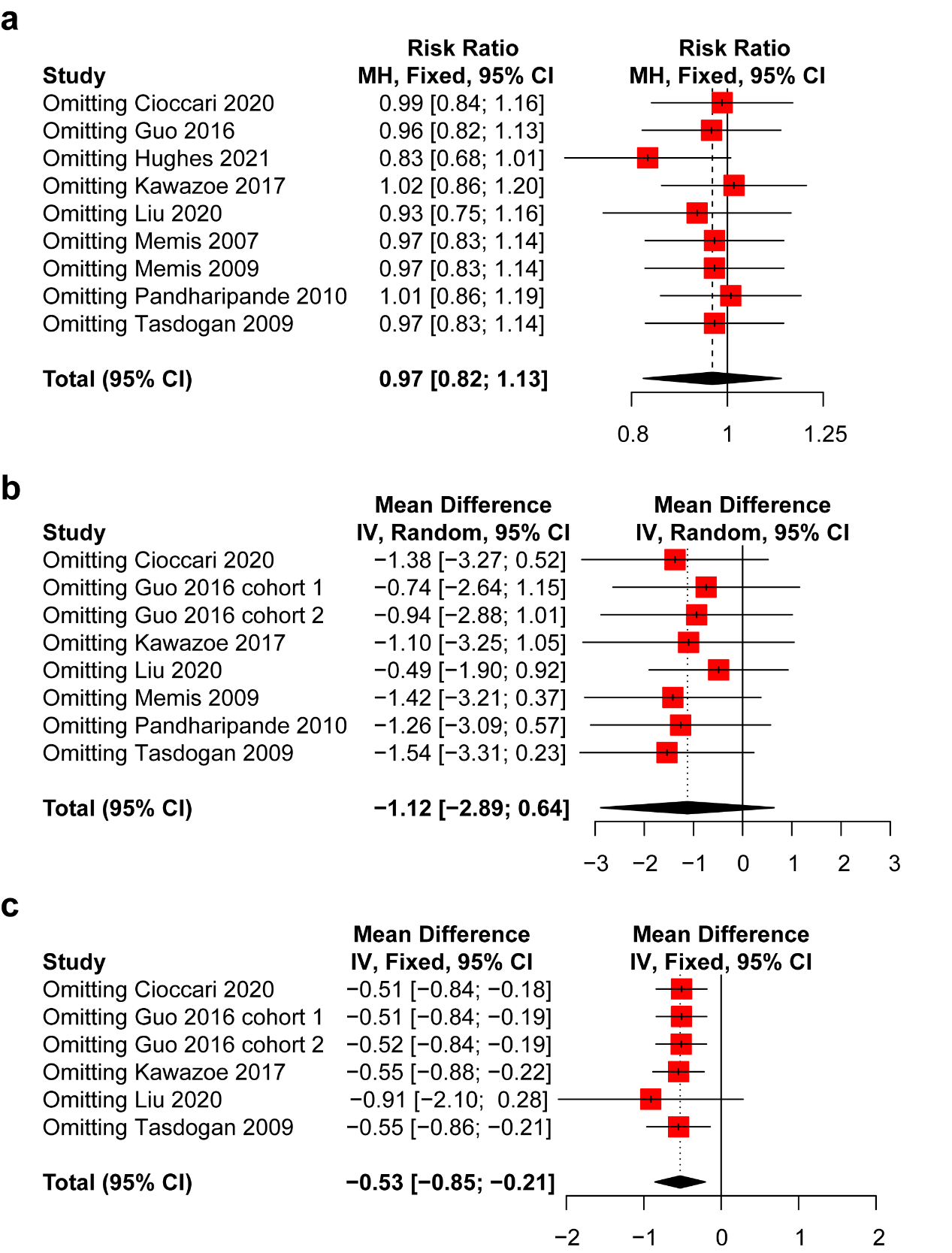


Figure 4: Sensitivity analysis; (a) mortality; (b) length of ICU stay; (c) duration of MV
