# Supplementary material for: The Effect of Dexmedetomidine as a Sedative Agent for Mechanically Ventilated Patients With Sepsis: A Systematic Review and Meta-Analysis
Source: Front Med (Lausanne). 2021 Dec 13;8:776882. doi: 10.3389/fmed.2021.776882 (PMC8711777; doi:10.3389/fmed.2021.776882)
Supplement: Supplementary file 5 [file Data_Sheet_5.docx]

**Supplementary File 5: Hemodynamic parameters**

| Study | Results |
| --- | --- |
| Hughes et al. 2021 | Hypotension: RR 1.01, 95%CI 0.85 to 1.19, P=0.95 |
| Cioccari et al. 2020 | Duration of vasopressor: MD 3.1, 95%CI -30.7 to 36.9,  P=0.72 |
| Pandharipande et al. 2010 | Needs increased vasopressor: RR 0.53, 95%CI 0.27 to 1.07,  P=0.08 |
| Tasdogan et al. 2009 | Needs vasopressor: RR 1.20, 95%CI 0.44 to 3.30, P=0.72 |
| Memis et al. 2009 | MAP: MD -2.00, 95%CI -10.37 to 6.37, P=0.64 |

RR: relative risk; MD: mean difference; MAP: mean arterial pressure
